# The post-marketing safety of venlafaxine: a real-world two-decade pharmacovigilance study using the FAERS database

**DOI:** 10.3389/fphar.2026.1737113

**Published:** 2026-01-21

**Authors:** Santosh Chokkakula, Hualiang Yang, Abeer A. Al-Masri, Yiquan Zhang, Bommireddy Naveen, Bing Yang

**Affiliations:** 1 Department of Microbiology, Chungbuk National University College of Medicine and Medical Research Institute, Cheongju, Republic of Korea; 2 Department of Pharmacy, Tianjin Central Hospital of Gynecology Obstetrics, Tianjin, China; 3 Department of Physiology, College of Medicine, King Saud University, Riyadh, Saudi Arabia; 4 School of Pharmacy, Tianjin Medical University, Tianjin, China; 5 Department of Chemical and Biological Engineering, Gachon University, Seongnam-Si, Gyeonggi-do, Republic of Korea; 6 Department of Cell Biology, College of Basic Medical Sciences, Tianjin Medical University, Tianjin, China; 7 Department of Public Health, International School, Krirk University, Bangkok, Thailand

**Keywords:** disproportionality analysis, FAERS, major depressive disorder, pharmacovigilance, venlafaxine

## Abstract

**Objective:**

Venlafaxine, a serotonin-norepinephrine reuptake inhibitor widely prescribed for major depressive disorder and related conditions, remains insufficiently characterized regarding its adverse event profile. This study aimed to comprehensively evaluate the post-marketing safety of venlafaxine using data from the U.S. Food and Drug Administration Adverse Event Reporting System (FAERS).

**Methods:**

The data were extracted from the FAERS database from the first quarter (Q1) 2004 to Q2 2025. Adverse drug events were analyzed using the Reporting Odds Ratio (ROR), Bayesian Confidence Propagation Neural Network (BCPNN), Proportional Reporting Ratio (PRR), and Multi-Item Gamma Poisson Shrinker (MGPS) methods.

**Results:**

A total of 47,325 venlafaxine-related adverse event reports were analyzed. Disproportionality analysis revealed significant signals for drug ineffectiveness (n = 2,108, ROR = 1.6, PRR = 1.6, χ^2^ = 483.4, IC = 0.7, EBGM = 1.6), drug hypersensitivity (n = 1,014; ROR = 4.9, PRR = 4.8, χ^2^ = 2998.7, IC = 2.2, EBGM = 4.7), and anxiety (n = 198, ROR = 4.3, PRR = 4.2, χ^2^ = 2202.0, IC = 2.0, EBGM = 4.1). Strongest signals (EBGM = 9.9) were observed for suicide attempt, agitated depression, and renal dysplasia. Tachycardia (EBGM = 8.3) was the most frequently reported adverse event among patients under 18 years, while emotional disorder (EBGM = 9.5) predominated in those aged 65 years and older. Most adverse events (39.3%) occurred within the first 30 days of venlafaxine therapy initiation.

**Conclusion:**

This pharmacovigilance analysis systematically identified significant safety signals associated with venlafaxine. The findings provide important evidence to support safer clinical use of venlafaxine and may assist in optimizing individualized therapeutic decisions in practice.

## 1 Introduction

Major depressive disorder (MDD) is a highly prevalent and often debilitating mental disorder associated with low mood, anhedonia, alterations in behavior, and emotional processing (American Psychiatric Association, 2022; Cao et al., 2019; Kaiser et al., 2015), and major impairments in social and occupational functioning (Fervaha et al., 2016; Stewart et al., 2003). An estimated 4% of the population encounters depression, including 5.7% of adults (4.6% among men and 6.9% among women), and 5.9% of adults aged 70 years and older. Approximately 332 million people in the world have depression (Global Burden of Disease GBD, 2021, 2024). Depression is about 1.5 times more common among women than among men. Worldwide, more than 10% of pregnant women and women who have just given birth experience depression (Woody et al., 2017). In 2021, an estimated 727,000 people lost their lives to suicide. Suicide is the third leading cause of death among 15–29-year-olds. According to a cross-national study, more than one in five people will experience at least one depression episode at some point in their lifetime (McGrath et al., 2020). In high-income countries, only about one-third of people with depression receive mental health treatment (Evans-Lacko et al., 2018).

Medication remains a central pillar in the management of MDD. Among the available antidepressants, venlafaxine, a serotonin-norepinephrine reuptake inhibitor (SNRI), represents one of the most extensively studied and prescribed drugs. In the United States alone, venlafaxine ranked 51st among all prescribed medications in 2023, with over 13.2 million prescriptions dispensed to nearly 3 million patients. This significant prescribing volume underscores the clinical importance of understanding its safety profile. Venlafaxine has received regulatory approval for the treatment of MDD in the United States, the United Kingdom, and across member states of the European Union (USFDA, 2009; European Medicines Age ncy, 2008; National Health Service, 2018). While typically recommended as a second-line intervention, venlafaxine is widely utilized, particularly among patients who exhibit inadequate response to first-line antidepressants (Singh and Saadabadi, 2024; Haddad et al., 2015; Kaplan, 2002). Its dose-dependent noradrenergic effects improve outcomes in treatment-resistant depression with faster onset and higher remission rates than SSRIs in nonresponders.

**FIGURE 1:**
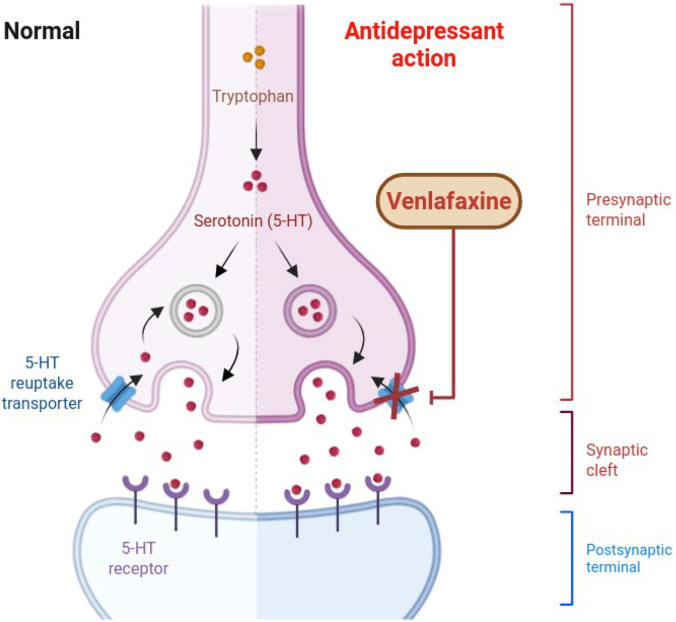
Mode of action of venlafaxine.

As an SNRI, venlafaxine works as a bicyclic drug, and it inhibits the reuptake of serotonin and norepinephrine, influencing central monoaminergic transmission (Figure 1) (Holliday and Benfield, 1995; Kent, 2000). However, despite decades of clinical use, no definitive dose escalation trials have reliably described an optimal dose response relationship that balances efficacy and tolerability (Kent, 2000; Hieronymus, 2019). This lack of rigorous dose-response characterization underscores the importance of large-scale real-world surveillance data, such as the U.S. Food and Drug Administration’s Adverse Event Reporting System (FAERS), to capture the spectrum of adverse events across diverse conditions.

Venlafaxine is associated with several well-documented safety concerns outlined in its prescribing information. The U.S product labeling carries a boxed warning noting that antidepressants increase the risk of suicidal thoughts and behavior in children, adolescents, and young adults (U.S. Food and Drug Administration, 2017). This is supported by a meta-analysis demonstrating an increased risk of suicidality in patients younger than 25 years treated with antidepressants (Stone et al., 2009; Hammad et al., 2006). Additional concerns in the labeling highlight risks of persistent hypertension, serotonin syndrome, and withdrawal symptoms upon discontinuation (Baldwin et al., 2007; Sternbach, 1991). Beyond these, case reports of overdose or intoxication have described severe complications, including persistent glucose-resistant hyperglycemia (Bekka and Eyer, 2022; Kobylianskii and Wu, 2021; Özdemir, 2021), acute cardiovascular toxicities such as myocardial injury and arrhythmias (Vinetti et al., 2011; Howell et al., 2007), and neurologic effects, including seizures and impaired consciousness (Khalifa et al., 1999).

Despite these known risks, most safety evidence for venlafaxine is derived from randomized controlled trials, which typically have limited durations, exclude many real-world populations, and many underdetect rare or delayed AEs. Moreover, real-world data regarding their incidence, severity, and temporal distribution remain limited. To bridge this gap, the present study utilizes the FAERS database, an extensive post-marketing surveillance resource that aggregates AE reports from diverse sources, including healthcare providers, patients, and pharmaceutical manufacturers, and regulatory stakeholders. Detailed parameters such as patient demographic, drug administration characteristics, timing, and nature of reported AEs, causality assessments, and clinical outcomes were systematically extracted from the analysis. To identify potential safety concerns, disproportionality analysis was employed to statistically evaluate the association between venlafaxine and specific AEs, comparing each observed drug-event pair to all others within the database. Heightened disproportionality metrics indicate that specific venlafaxine-AE combinations are occurring more frequently than expected by chance, suggesting a potential safety signal for further investigation. Through this approach, the current study aims to clarify the real-world safety profile of venlafaxine, detect novel risk signals, and provide actionable evidence to support clinicians and pharmacists in optimizing therapeutic decisions and ensuring patient safety.

## 2 Materials and methods

### 2.1 Data source and study design

This retrospective pharmacovigilance study analyzed adverse event reports from the FAERS database. FAERS is a publicly accessible, spontaneous reporting database collecting post-marketing safety data submitted by healthcare professionals, consumers, and pharmaceutical manufacturers. Data spanning from the first quarter of 2004 to the second quarter of 2025 were downloaded in ASCII format from the FDA website. The investigation focused on venlafaxine, and cases were identified using the generic drug name “Venlafaxine,” “Venlafaxine Hcl,” “Venlafaxine Hydrochloride,” and the brand names “Effexor,” “Effexor xr,” “Venbysi xr”. All adverse events were codified according to the Medical Dictionary for Regulatory Activities (MedDRA) Version 28.1, which provides a standardized hierarchical classification of adverse events from detailed Preferred Terms (PTs) to aggregate System Organ Classes (SOCs).

### 2.2 Data preprocessing and quality control

**FIGURE 2:**
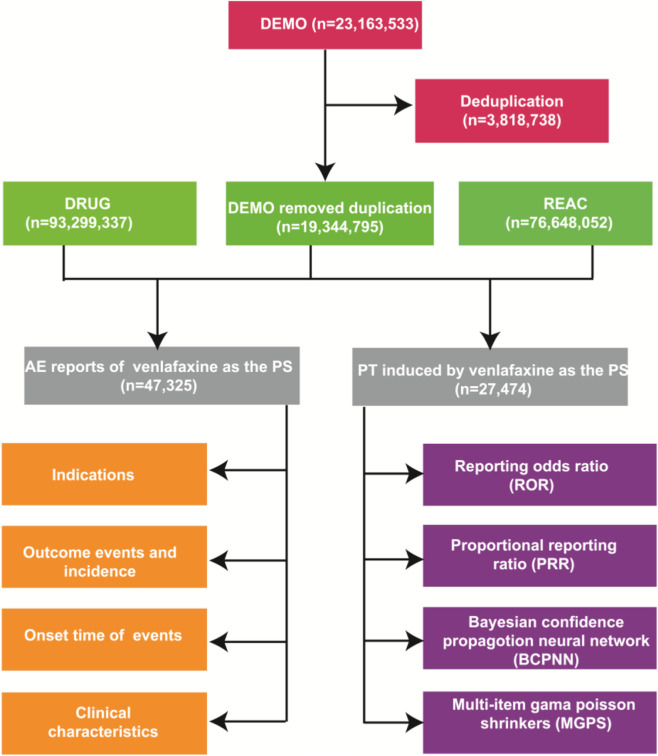
The flow diagram of selecting and analyzing venlafaxine-related ADEs from FAERS database.

Data cleaning was performed using SAS software version 9.4 to ensure the reliability of analyses. The FAERS database often contains duplicate case reports and inconsistent or missing data due to its voluntary reporting nature. Deduplication was conducted following FDA guidelines by sorting reports based on unique identifiers, including CASEID, PRIMARYID, and FDA_DT (report date). When multiple reports shared the same CASEID, only the latest report (determined by the highest FDA_DT) was retained. In cases where the CASEID and FDA_DT were identical, the submission with the highest PRIMARYID was kept, preserving the most current information. After deduplication, the cleaned demographic (DEMO), drug (DRUG), and adverse reaction (REAC) datasets were merged. Analysis was restricted to cases where venlafaxine was declared the primary suspect drug to reduce confounding effects. Time-to-onset calculations were performed by subtracting the start date of venlafaxine therapy from the adverse event occurrence date. Inconsistent cases, such as those with AE dates preceding drug start or missing dates, were excluded from analysis. The data processing workflow is shown in Figure 2.

### 2.3 Disproportionality signal detection methods

To detect potential associations between venlafaxine and reported adverse events, disproportionality analysis, a cornerstone method in pharmacovigilance, was employed using four complementary algorithms, such as Reporting Odds Ratio (ROR), Proportional Reporting Ratio (PRR), Bayesian Confidence Propagation Neural Network (BCPNN), and Multi-Item Gamma Poisson Shrinker (MGPS). The two-by-two table for understanding these methods is provided in Supplementary Table S1. These methods evaluate whether a drug-event pair occurs more frequently than expected compared to the full FAERS database, indicating potential safety signals. Frequentist methods (ROR and PRR) provide sensitivity in detecting commonly reported events, while Bayesian approaches (BCPNN and MGPS) better adjust for low counts and reduce false positives. The combination of these diverse methods enhances the robustness and reliability of signal detection.

### 2.4 Subgroup analysis

To elucidate variations in AE patterns across patient populations, subgroup analyses were conducted according to key demographic and clinical variables. Patients were categorized by age (<18 years, 18–60 years, and >60 years), sex (male and female), and geographic region to assess differences in AE distributions among these subgroups. This stratified analysis facilitated the identification of demographic trends and potential vulnerability profiles associated with Venlafaxine exposure. In addition, a time-to-onset analysis was performed to characterize the temporal relationship between the initiation of venlafaxine therapy and the manifestation of AEs, providing insights into latency periods and the progression of drug-related safety signals.

### 2.5 Signal criteria and statistical analysis

Signals were defined according to established international thresholds as described in Supplementary Table S2. For ROR and PRR, signals required a minimum of three case reports, with the lower bound of the 95% confidence interval exceeding 1. PRR further required a minimum ratio value of 2 to consider a signal noteworthy. The BCPNN method flagged signals when the lower limit of the Information Component’s 95% credibility interval (IC025) surpassed zero, consistent with World Health Organization guidelines. The MGPS criterion required the lower bound of the Empirical Bayes Geometric Mean’s 95% confidence interval (EBGM05) to exceed 2, providing a conservative measure for rare events. These criteria were applied using SAS and SQL to process and analyze large-scale datasets efficiently. This multipronged approach allowed for balanced sensitivity and specificity in identifying venlafaxine-related adverse event signals.

Because disproportionality analyses evaluate thousands of drug–event combinations, multiplicity is an inherent concern. Classical statistical corrections are not routinely applied in pharmacovigilance signal detection; instead, shrinkage-based methods (MGPS, BCPNN) and minimum case-count thresholds help reduce random fluctuation. Despite these built-in protections, residual false-positive signals may still occur and should be interpreted cautiously.

## 3 Results

### 3.1 Basic information on AERs

A comprehensive analysis of venlafaxine-related adverse event reports (AERs) identified 47,325 cases in the study period, which is shown in Figure 2. Demographic analysis revealed a significant gender disparity, with female patients accounting for 63.8% of reports compared to 25.0% in males. The age distribution demonstrated the following pattern: <18 years (1.3%), 18–44 years (26.8%), 45–64 years (24.0%), >64 years (12.1%), with 35.8% of reports containing unspecified age data. The mean age of reported cases was 47.4 years. Temporal analysis indicated 2013 as the peak reporting year (11.2% of total reports). Primary reporters consisted predominantly of consumers (53.1%), followed by physicians (26.3%) and other healthcare professionals (12.3%). Geographically, the United States contributed the majority of reports (57.8%), followed by the United Kingdom (15.2%). Clinical outcomes analysis revealed hospitalization (initial or prolonged) as the most frequent serious outcome (52.7%), with deaths reported in 16.7% of cases. Temporal pattern analysis showed 39.3% of adverse events occurred within 30 days of treatment initiation, though the mean time to event onset was 493.9 days. The mean patient weight across reported cases was 79.4 kg, and all these demographic results are shown in Table 1.

**TABLE 1:** Demographic and clinical characteristics of adverse event reports for venlafaxine in the FAERS database (2004–2025).

Characteristic	Number of cases (%)
Gender	
Female	30,216 (63.8)
Male	11,810 (25.0)
Unknown	5,299 (11.2)
Age	
<18	610 (1.3)
18–44	12,692 (26.8)
45–64	11,358 (24.0)
>64	5,706 (12.1)
Unknown	16,959 (35.8)
Age (quantitative)	
N (missing)	31,676 (15.649)
Mean (SD)	47.4 (177.96)
Median (Q1, Q3)	47.0 (32.60)
Min, Max	0, 23124
Reporting year	
2011	5 (0.0)
2012	1,063 (2.3)
2013	5,283 (11.2)
2014	3,361 (7.1)
2015	3,453 (7.3)
2016	3,326 (7.0)
2017	3,474 (7.4)
2018	4,413 (9.3)
2019	5,060 (10.7)
2020	3,737 (7.9)
2021	3,005 (6.4)
2022	2,967 (6.3)
2023	3,207 (6.8)
2024	3,190 (6.8)
2025	1,680 (3.6)
Reporter	
Consumer	22,123 (53.1)
Lawyer	199 (0.5)
Not specified	5,674 (0.0)
Other health-professional	5,140 (12.3)
Physician	3,215 (7.7)
Medical doctor	10,974 (26.3)
Reporting countries (Top 5)	
Canada	1,320 (3.6)
Germany	4,411 (12.0)
France	4,178 (11.4)
United Kingdom	5,604 (15.2)
United States	21,245 (57.8)
Outcome	
Congenital anomaly	928 (5.2)
Death	2,975 (16.7)
Disability	2,194 (12.3)
Hospitalization - initial or prolonged	9,372 (52.7)
Life-threatening	2,324 (13.1)
Onset time	
0–30 d	1979 (39.3)
31–60 d	404 (8.0)
61–90 d	192 (3.8)
91–120 d	140 (2.8)
121–150 d	139 (2.8)
151–180 d	91 (1.8)
181–360 d	1,021 (20.3)
>360 d	1,064 (21.2)
Time of occurrence of adverse events	
N (missing)	5,030 (0)
Mean (SD)	493.9 (1153.2)
Median (Q1, Q3)	72.5 (10.277)
Min, Max	110, 743
Weight (KG)	
N (missing)	15,272 (32.053)
Mean (SD)	79.4 (42.30)
Median (Q1, Q3)	73.0 (60.93)
Min, Max	0.400

### 3.2 Onset time of adverse events

**FIGURE 3:**
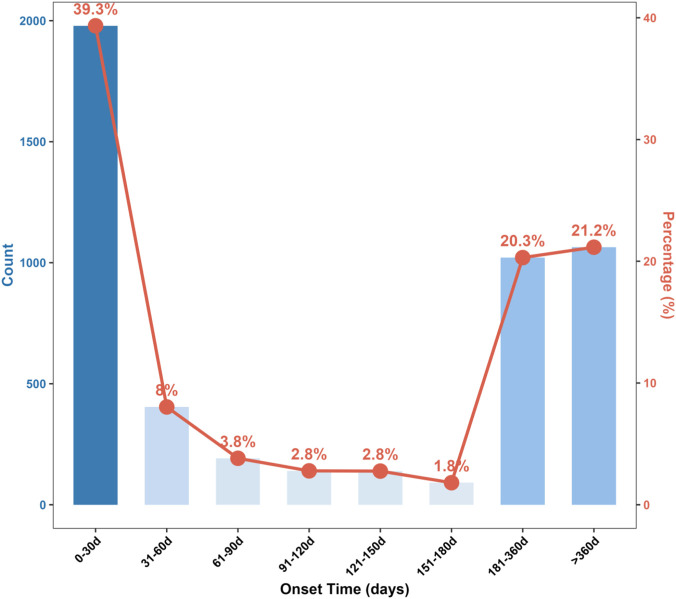
Onset time distribution of venlafaxine-related adverse events (AEs).

The onset time of adverse events related to venlafaxine was evaluated to understand the temporal distribution of reported reactions following drug administration (Table 1; Figure 3).

The mean onset time was 493.9 days (SD = 1153.2), while the median onset was 72.5 days (interquartile range: 10–277 days). The shortest observed onset was 1 day, and the longest was 10,743 days, indicating considerable variability in the timing of adverse event occurrence.

The distribution of onset times, as visualized in Figure 3, revealed a bimodal pattern. The majority of adverse events (39.3%) occurred within the first 30 days after venlafaxine initiation, as seen by the prominent initial bar in the graph. Event frequency then declined with increasing duration. The 8.0% of events were reported between 31 and 60 days, 3.8% between 61 and 90 days, and approximately 2.8% each for both 91–120 days and 121–150 days, with a low of 1.8% for 151–180 days. Notably, a substantial proportion of events also occurred at later intervals, such as 20.3% between 181 and 360 days and 21.2% beyond 360 days of drug exposure.

### 3.3 Distribution of the AERs at the preferred terms (PT) level

As presented in Table 2, the top 30 Preferred Terms (PTs) ranked by signal frequency revealed drug ineffective (n = 2,108; ROR = 1.6, PRR = 1.6, IC = 0.7, EBGM = 1.6) as the most frequently reported event. The subsequent highest-frequency signals included drug hypersensitivity (n = 1,014; ROR = 4.9, PRR = 4.8, IC = 2.2, EBGM = 4.7), anxiety (n = 918; ROR = 4.3, PRR = 4.2, IC = 2.0, EBGM = 4.1), dizziness (n = 824; ROR = 2.6, PRR = 2.5, IC = 1.3, EBGM = 2.5), and completed suicide (n = 786; ROR = 11.0, PRR = 10.7, IC = 3.4, EBGM = 10.4). Except for drug ineffectiveness, all high-frequency PTs correspond with known adverse reactions documented in venlafaxine’s prescribing information.

**TABLE 2:** The top 30 PTs ranked by the frequency of positive signals.

SOC	PT	Case reports (n)	ROR (95% CI)	PRR (95% CI)	χ2	IC (IC025)	EBGM (EBGM05)
General disorders and administration site conditions	Drug ineffective	2,108	1.6 (1.6, 1.7)	1.6 (1.5, 1.7)	483.4	0.7 (0.6)	1.6 (1.5)
Immune system disorders	Drug hypersensitivity	1,014	4.9 (4.6, 5.2)	4.8 (4.5, 5.0)	2998.7	2.2 (2.2)	4.7 (4.5)
Psychiatric disorders	Anxiety	918	4.3 (4.0, 4.6)	4.2 (3.9, 4.4)	2202.0	2.0 (2.0)	4.1 (3.9)
Nervous system disorders	Dizziness	824	2.6 (2.4, 2.8)	2.5 (2.4, 2.7)	775.1	1.3 (1.2)	2.5 (2.4)
Psychiatric disorders	Completed suicide	786	11.0 (10.2, 11.8)	10.7 (10.0, 11.5)	6791.1	3.4 (3.3)	10.4 (9.9)
Nervous system disorders	Headache	636	1.6 (1.5, 1.7)	1.6 (1.5, 1.7)	136.8	0.7 (0.5)	1.6 (1.5)
Gastrointestinal disorders	Nausea	596	1.4 (1.3, 1.5)	1.4 (1.3, 1.5)	66.5	0.5 (0.4)	1.4 (1.3)
General disorders and administration site conditions	Withdrawal syndrome	561	23.5 (21.6, 25.6)	23.0 (21.2, 25.1)	11,378	4.5 (4.4)	21.8 (20.3)
General disorders and administration site conditions	Feeling abnormal	535	3.6 (3.3, 3.9)	3.6 (3.3, 3.9)	978.7	1.8 (1.7)	3.5 (3.3)
Psychiatric disorders	Depression	532	4.2 (3.9, 4.6)	4.2 (3.8, 4.5)	1271.6	2.0 (1.9)	4.1 (3.8)
General disorders and administration site conditions	Malaise	524	1.9 (1.7, 2.0)	1.9 (1.7, 2.0)	209.8	0.9 (0.8)	1.9 (1.7)
General disorders and administration site conditions	Fatigue	501	1.0 (1.0, 1.1)	1.0 (1.0, 1.1)	0.7	0.1 (−0.1)	1.0 (1.0)
Injury, poisoning and procedural complications	Toxicity to various agents	480	4.4 (4.0, 4.8)	4.4 (4.0, 4.8)	1235.0	2.1 (2.0)	4.3 (4.0)
General disorders and administration site conditions	Drug withdrawal syndrome	455	9.9 (9.0, 10.9)	9.8 (8.9, 10.7)	3527.0	3.3 (3.1)	9.5 (8.8)
General disorders and administration site conditions	Drug interaction	449	5.9 (5.3, 6.4)	5.8 (5.3, 6.3)	1760.1	2.5 (2.4)	5.7 (5.3)
Psychiatric disorders	Insomnia	446	3.4 (3.1, 3.7)	3.4 (3.1, 3.7)	741.9	1.7 (1.6)	3.3 (3.1)
Injury, poisoning and procedural complications	Foetal exposure during pregnancy	406	6.1 (5.5, 6.7)	6.0 (5.5, 6.6)	1680.0	2.6 (2.4)	5.9 (5.5)
Psychiatric disorders	Drug abuse	402	5.1 (4.7, 5.7)	5.1 (4.6, 5.6)	1310.8	2.3 (2.2)	5.0 (4.6)
Psychiatric disorders	Agitation	385	7.1 (6.4, 7.9)	7.0 (6.4, 7.8)	1967.5	2.8 (2.7)	6.9 (6.3)
General disorders and administration site conditions	Asthenia	363	1.3 (1.2, 1.4)	1.3 (1.2, 1.4)	21.4	0.3 (0.2)	1.3 (1.2)
Psychiatric disorders	Suicidal ideation	349	7.8 (7.0, 8.7)	7.7 (6.9, 8.6)	2009.4	2.9 (2.8)	7.5 (6.9)
Skin and subcutaneous tissue disorders	Hyperhidrosis	339	5.4 (4.8, 6.0)	5.3 (4.8, 5.9)	1181.9	2.4 (2.2)	5.3 (4.8)
General disorders and administration site conditions	Death	338	0.3 (0.3, 0.3)	0.3 (0.3, 0.4)	516.4	−1.7 (−1.8)	0.3 (0.3)
Injury, poisoning and procedural complications	Intentional overdose	335	8.7 (7.8, 9.7)	8.6 (7.7, 9.6)	2220.9	3.1 (2.9)	8.4 (7.7)
Injury, poisoning and procedural complications	Overdose	327	2.7 (2.4, 3.0)	2.7 (2.4, 3.0)	340.2	1.4 (1.3)	2.7 (2.4)
General disorders and administration site condition	Crying	319	15.0 (13.4, 16.7)	14.8 (13.3, 16.6)	4003.1	3.9 (3.7)	14.2 (12.9)
Nervous system disorders	Tremor	294	3.9 (3.5, 4.4)	3.9 (3.5, 4.4)	634.1	2.0 (1.8)	3.9 (3.5)
Cardiac disorders	Cardiac arrest	292	4.9 (4.4, 5.5)	4.9 (4.3, 5.4)	884.0	2.3 (2.1)	4.8 (4.3)
General disorders and administration site conditions	Condition aggravated	290	1.3 (1.2, 1.5)	1.3 (1.2, 1.5)	22.2	0.4 (0.2)	1.3 (1.2)
Nervous system disorders	Somnolence	288	3.0 (2.6, 3.3)	2.9 (2.6, 3.3)	367.9	1.6 (1.4)	2.9 (2.7)

As detailed in Table 3, highest signal intensity was identified for suicide attempt (n = 262; ROR = 10.3, PRR = 10.2, IC = 3.3, EBGM = 9.9), agitated depression (n = 7; ROR = 30.7, PRR = 32.6, IC = 4.8, EBGM = 9.9), renal dysplasia (n = 6; ROR = 43.4, PRR = 46.5, IC = 5.2, EBGM = 9.9), bipolar I disorder (n = 18; ROR = 13.7, PRR = 14.0, IC = 3.7, EBGM = 9.8), and parotid gland enlargement (n = 7; ROR = 29.2, PRR = 31.1, IC = 4.7, EBGM = 9.8). Despite lower case counts, these PTs demonstrated substantially higher signal intensities, suggesting potential novel adverse reaction associations warranting further clinical investigation.

**TABLE 3:** Top 30 PTs ranked by the intensity of positive signals.

SOC	PT	Case reports (n)	ROR (95% CI)	PRR (95% CI)	χ2	IC (IC025)	EBGM (EBGM05)
Psychiatric disorders	Suicide attempt	262	10.3 (9.1, 11.7)	10.2 (9.1, 11.6)	2140.5	3.3 (3.2)	9.9 (8.9)
Psychiatric disorders	Agitated depression	7	30.7 (14.4, 65.3)	32.6 (15.3, 69.4)	178.5	4.8 (3.7)	9.9 (5.6)
Congenital, familial and genetic disorders	Renal dysplasia	6	43.4 (19.0, 98.9)	46.5 (20.4, 106)	215.1	5.2 (4.1)	9.9 (5.3)
Psychiatric disorders	Bipolar I disorder	18	13.7 (8.6, 21.8)	14.0 (8.8, 22.4)	200.3	3.7 (3.0)	9.8 (6.8)
Gastrointestinal disorders	Parotid gland enlargement	7	29.2 (13.7, 62.2)	31.1 (14.6, 66.1)	170.0	4.7 (3.6)	9.8 (5.5)
Congenital, familial and genetic disorders	Abnormal palmar/plantar creases	6	42.4 (18.6, 96.6)	45.4 (19.9, 103)	210.4	5.2 (4.0)	9.8 (5.3)
Investigations	Acoustic stimulation tests abnormal	6	41.5 (18.2, 94.5)	44.5 (19.5, 101)	205.8	5.2 (4.0)	9.8 (5.3)
Respiratory, thoracic and mediastinal disorders	Yawning	16	14.1 (8.6, 23.1)	14.5 (8.8, 23.8)	183.4	3.7 (3.0)	9.7 (6.5)
Psychiatric disorders	Obsessive thoughts	11	17.2 (9.5, 31.3)	17.9 (9.9, 32.6)	155.7	4.0 (3.2)	9.7 (6.0)
Psychiatric disorders	Mania	86	10.4 (8.4, 12.9)	10.4 (8.4, 12.9)	711.6	3.3 (3.0)	9.6 (8.1)
Psychiatric disorders	Homicidal ideation	19	13.0 (8.2, 20.4)	13.3 (8.5, 20.9)	199.6	3.6 (3.0)	9.6 (6.7)
Renal and urinary disorders	Urge incontinence	7	27.4 (12.9, 58.3)	29.2 (13.7, 62.0)	159.1	4.6 (3.5)	9.6 (5.4)
Respiratory, thoracic and mediastinal disorders	Immature respiratory system	5	84.4 (33.4, 214)	90.9 (35.9, 230)	334.6	6.1 (4.8)	9.6 (4.9)
General disorders and administration site	Drug withdrawal syndrome	455	9.9 (9.0, 10.9)	9.8 (8.9, 10.7)	3527.0	3.3 (3.1)	9.5 (8.8)
Renal and urinary disorders	Microalbuminuria	6	36.1 (15.9, 81.8)	38.6 (17.0, 87.7)	178.7	5.0 (3.8)	9.4 (5.1)
Eye disorders	Anisocoria	6	35.4 (15.6, 80.3)	37.9 (16.7, 86.0)	175.3	5.0 (3.8)	9.4 (5.0)
Investigations	False positive investigation result	14	13.9 (8.2, 23.5)	14.3 (8.4, 24.3)	157.1	3.7 (3.0)	9.3 (6.1)
Nervous system disorders	Hypoxic-ischemic encephalopathy	13	14.5 (8.4, 25.1)	15.0 (8.7, 26.0)	152.8	3.8 (3.0)	9.3 (6.0)
Blood and lymphatic system disorders	Warm autoimmune haemolytic anemia	5	60.7 (24.4, 151)	65.6 (26.3, 163)	245.5	5.7 (4.4)	9.2 (4.7)
Nervous system disorders	Generalised tonic-clonic seizure	113	9.7 (8.0, 11.7)	9.7 (8.1, 11.7)	858.2	3.2 (3.0)	9.1 (7.8)
Nervous system disorders	Apraxia	12	14.3 (8.1, 25.4)	14.9 (8.4, 26.3)	138.8	3.8 (2.9)	9.0 (5.7)
Renal and urinary disorders	Bladder irritation	8	19.5 (9.7, 39.3)	20.6 (10.2, 41.5)	127.5	4.2 (3.2)	9.0 (5.2)
General disorders and administration site conditions	Alcohol interaction	20	11.5 (7.4, 17.9)	11.8 (7.6, 18.3)	182.9	3.5 (2.8)	8.9 (6.2)
Psychiatric disorders	Tearfulness	16	12.3 (7.5, 20.1)	12.6 (7.7, 20.7)	156.6	3.6 (2.8)	8.8 (5.9)
Nervous system disorders	Cerebral haemorrhage neonatal	5	46.7 (18.9, 115)	50.6 (20.5, 125)	190.3	5.3 (4.0)	8.8 (4.5)
Congenital, familial and genetic disorders	Hemimegalencephaly	4	1,063 (198, 5,705)	984.3 (183, 5,281)	1572.3	8.6 (6.8)	8.8 (4.2)
Congenital, familial and genetic disorders	Neuronal migration disorder	4	1,063 (198, 5,705)	984.3 (183, 5,281)	1572.3	8.6 (6.8)	8.8 (4.2)
Nervous system disorders	Sleep paralysis	13	13.1 (7.5, 22.6)	13.5 (7.8, 23.4)	135.8	3.6 (2.8)	8.7 (5.6)
Nervous system disorders	Cerebellar haematoma	6	26.9 (11.9, 60.8)	28.9 (12.8, 65.2)	132.4	4.6 (3.4)	8.7 (4.7)
Congenital, familial and genetic disorders	Neonatal disorder	6	27.7 (12.3, 62.6)	29.7 (13.2, 67.1)	136.4	4.6 (3.5)	8.7 (4.7)

### 3.4 Sex-based subgroup analysis

A sex-based subgroup analysis was conducted to explore differences in adverse event reporting associated with venlafaxine. PTs meeting all four statistical detection criteria were identified separately for males and females, as summarized in Table 4.

**TABLE 4:** Signal strength of reports of venlafaxine at the preferred term level in the FAERS database grouped by sex.

Sex	PT	Case reports (n)	ROR (95% CI)	PRR (95% CI)	χ2	IC (IC025)	EBGM (EBGM05)
Female	Nightmare	96	8.7 (7.1, 10.6)	8.6 (7.1, 10.6)	640.1	3.1 (2.8)	9.7 (8.2)
	Disturbance in attention	157	8.5 (7.3, 10.0)	8.4 (7.2, 9.9)	1014.7	3.1 (2.8)	9.6 (8.5)
	Suicide attempt	133	8.3 (7.0, 9.9)	8.3 (7.0, 9.9)	840.5	3.0 (2.8)	9.4 (8.2)
	Intentional overdose	211	8.2 (7.1, 9.4)	8.1 (7.1, 9.4)	1296.8	3.0 (2.8)	9.3 (8.4)
	Foetal exposure during pregnancy	150	8.1 (6.9, 9.5)	8.0 (6.8, 9.5)	909.1	3.0 (2.7)	9.2 (8.0)
	Agitation	242	7.9 (7.0, 9.0)	7.8 (6.9, 9.0)	1421.6	2.9 (2.8)	9.1 (8.2)
	Anger	114	8.1 (6.7, 9.7)	8.0 (6.7, 9.7)	695.2	3.0 (2.7)	9.1 (7.8)
	Suicidal ideation	212	7.8 (6.8, 8.9)	7.7 (6.7, 8.9)	1219.0	2.9 (2.7)	8.9 (7.9)
	Irritability	149	7.9 (6.7, 9.2)	7.8 (6.6, 9.2)	873.2	2.9 (2.7)	8.9 (7.8)
	Depressed mood	183	7.6 (6.6, 8.8)	7.5 (6.5, 8.8)	1024.0	2.9 (2.7)	8.7 (7.7)
	Coma	137	7.7 (6.5, 9.1)	7.6 (6.4, 9.1)	777.5	2.9 (2.7)	8.7 (7.6)
	Intentional self-injury	57	7.8 (6.0, 10.1)	7.8 (6.0, 10.1)	333.4	2.9 (2.6)	8.5 (6.8)
	Drug abuse	240	6.7 (5.9, 7.6)	6.6 (5.8, 7.6)	1131.6	2.7 (2.5)	7.7 (6.9)
	Drug interaction	264	6.6 (5.8, 7.4)	6.5 (5.8, 7.4)	1214.7	2.7 (2.5)	7.6 (6.8)
	Electrocardiogram qt prolonged	81	6.6 (5.3, 8.2)	6.6 (5.3, 8.2)	379.2	2.7 (2.4)	7.4 (6.2)
	Activities of daily living impaired	79	6.4 (5.1, 8.0)	6.4 (5.1, 8.0)	357.4	2.7 (2.3)	7.2 (6.0)
	Feelings of worthlessness	72	126.2 (97.5, 163)	125.7 (97.2, 163)	7146.8	6.6 (6.3)	65.8 (54.3)
	Feeling guilty	75	106.4 (82.9, 136)	105.9 (82.7, 136)	6460.6	6.4 (6.1)	61.9 (51.3)
	Nervousness	122	6.0 (5.0, 7.2)	6.0 (5.0, 7.2)	504.1	2.6 (2.3)	6.9 (6.0)
	Cardiac arrest	180	5.9 (5.1, 6.8)	5.8 (5.0, 6.8)	711.7	2.5 (2.3)	6.8 (6.0)
Male	Foetal exposure during pregnancy	163	12.3 (10.5, 14.4)	12.0 (10.3, 14.3)	1632.5	3.6 (3.3)	8.9 (7.8)
	Completed suicide	263	11.6 (10.2, 13.1)	11.2 (9.9, 13.1)	2,420.0	3.5 (3.3)	8.3 (7.5)
	Suicide attempt	76	11.7 (9.3, 14.7)	11.6 (9.3, 14.7)	730.5	3.5 (3.2)	8.3 (6.9)
	Erectile dysfunction	117	9.5 (7.9, 11.4)	9.3 (7.8, 11.3)	864.1	3.2 (2.9)	6.9 (5.9)
	Disturbance in attention	67	8.9 (7.0, 11.3)	8.8 (6.9, 11.3)	462.4	3.1 (2.8)	6.4 (5.2)
	Suicidal ideation	95	8.0 (6.5, 9.7)	7.9 (6.4, 9.7)	567.1	3.0 (2.7)	5.8 (4.9)
	Depressed mood	61	8.0 (6.2, 10.3)	8.0 (6.2, 10.3)	370.4	3.0 (2.6)	5.8 (4.7)
	Intentional overdose	72	7.9 (6.2, 9.9)	7.8 (6.2, 9.9)	425.9	3.0 (2.6)	5.7 (4.7)
	Irritability	57	7.4 (5.7, 9.6)	7.3 (5.7, 9.6)	312.4	2.9 (2.5)	5.3 (4.3)
	Anger	56	7.1 (5.5, 9.3)	7.1 (5.4, 9.2)	292.2	2.8 (2.4)	5.1 (4.1)
	Agitation	116	6.7 (5.6, 8.1)	6.6 (5.5, 8.1)	555.3	2.7 (2.5)	5.0 (4.3)
	Coma	61	6.8 (5.3, 8.8)	6.8 (5.3, 8.8)	301.9	2.8 (2.4)	5.0 (4.0)
	Toxicity to various agents	147	5.6 (4.8, 6.6)	5.5 (4.7, 6.6)	546.9	2.5 (2.2)	4.2 (3.6)
	Drug withdrawal syndrome	90	5.4 (4.4, 6.7)	5.3 (4.4, 6.6)	318.4	2.4 (2.1)	4.0 (3.4)
	Maternal exposure during pregnancy	54	60.8 (46.1, 80.2)	60.3 (45.8, 80.0)	2,949.3	5.8 (5.4)	30.9 (24.7)
	Drug interaction	128	5.2 (4.4, 6.2)	5.2 (4.4, 6.2)	430.6	2.4 (2.1)	3.9 (3.4)
	Anxiety	221	4.7 (4.1, 5.4)	4.6 (4.0, 5.4)	621.7	2.2 (2.0)	3.5 (3.1)
	Drug abuse	146	4.7 (4.0, 5.5)	4.6 (3.9, 5.5)	413.0	2.2 (2.0)	3.5 (3.0)
	Drug hypersensitivity	154	4.7 (4.0, 5.5)	4.6 (3.9, 5.5)	432.8	2.2 (2.0)	3.5 (3.0)
	Aggression	74	4.8 (3.8, 6.0)	4.7 (3.8, 6.0)	217.9	2.2 (1.9)	3.5 (2.9)

Among female patients, PTs with more than 50 reported cases included nightmare, disturbance in attention, suicide attempt, intentional overdose, and foetal exposure during pregnancy, agitation, anger, suicidal ideation, irritability, depressed mood, coma, intentional self-injury, drug abuse, drug interaction, and electrocardiogram QT prolongation, impaired activities of daily living, feelings of worthlessness, feeling guilty, nervousness, and cardiac arrest. The highest EBGM values were observed for feelings of worthlessness (EBGM = 65.8) and feeling guilty (EBGM = 61.9), indicating strong signal strength within the female subgroup (Table 4).

Among male patients, PTs with more than 50 reported cases included foetal exposure during pregnancy, completed suicide, suicide attempt, erectile dysfunction, disturbance in attention, suicidal ideation, depressed mood, intentional overdose, irritability, anger, agitation, coma, toxicity to various agents, drug withdrawal syndrome, maternal exposure during pregnancy, drug interaction, anxiety, drug abuse, drug hypersensitivity, and aggression. The highest EBGM values were identified for maternal exposure during pregnancy (EBGM = 30.9) and foetal exposure during pregnancy (EBGM = 8.9), showing significant signal intensity among male patients (Table 4).

### 3.5 Age-based subgroup analysis in different PT groups

An age-stratified subgroup analysis was conducted to identify variations in venlafaxine-associated adverse event reporting across different age categories (<18, 18–44, 45–64, and >64 years). PTs demonstrating statistical significance across all four signal detection methods within each age group are summarized in Table 5.

**TABLE 5:** Signal strength of venlafaxine at the preferred term class level in the FAERS database grouped by age.

Age	PT	Case reports (n)	ROR (95% CI)	PRR (95% CI)	χ2	IC (IC025)	EBGM (EBGM05)
<18	Tachycardia	12	12.1 (6.9, 21.4)	11.7 (6.8, 20.2)	122.0	3.5 (2.8)	8.3 (5.3)
	Somnolence	18	10.3 (6.4, 16.5)	9.8 (6.3, 15.3)	145.7	3.3 (2.6)	7.9 (5.5)
18–44	Feeling guilty	12	15.7 (8.9, 27.7)	15.6 (8.8, 27.6)	162.2	3.9 (3.1)	9.7 (6.2)
	Drug withdrawal syndrome	102	10.4 (8.5, 12.6)	10.2 (8.4, 12.4)	824.5	3.3 (3.0)	9.5 (8.1)
	Restless legs syndrome	26	10.3 (7.0, 15.2)	10.2 (7.0, 15.1)	213.2	3.3 (2.8)	8.5 (6.2)
	Depersonalization/derealisation disorder	13	12.3 (7.1, 21.2)	12.2 (7.1, 21.1)	133.6	3.6 (2.8)	8.5 (5.5)
	Hypoglycemia	56	9.3 (7.1, 12.1)	9.2 (7.1, 12.0)	402.0	3.2 (2.8)	8.4 (6.8)
	Psychiatric symptom	17	10.9 (6.8, 17.6)	10.9 (6.8, 17.6)	151.6	3.4 (2.7)	8.3 (5.7)
	Decreased interest	15	11.0 (6.6, 18.3)	11.0 (6.6, 18.3)	135.6	3.4 (2.7)	8.2 (5.5)
	Galactorrhoea	18	10.4 (6.6, 16.6)	10.4 (6.6, 16.6)	151.9	3.3 (2.7)	8.1 (5.6)
	Apathy	48	9.0 (6.7, 11.9)	8.9 (6.7, 11.8)	329.7	3.1 (2.7)	8.0 (6.4)
	Gestational diabetes	29	9.3 (6.5, 13.5)	9.3 (6.4, 13.4)	211.6	3.2 (2.6)	8.0 (5.9)
45–64	Shock hemorrhagic	12	15.8 (9.0, 27.8)	15.8 (9.0, 27.7)	166.9	3.9 (3.1)	9.9 (6.3)
	Bruxism	12	14.5 (8.3, 25.5)	14.5 (8.3, 25.5)	152.0	3.8 (3.0)	9.4 (6.0)
	Dependence	10	15.8 (8.5, 29.2)	15.8 (8.5, 29.1)	139.9	3.9 (3.0)	9.2 (5.7)
	Colitis microscopic	17	12.1 (7.5, 19.5)	12.1 (7.5, 19.4)	173.1	3.6 (2.9)	9.1 (6.2)
	Mydriasis	13	13.2 (7.7, 22.6)	13.1 (7.7, 22.6)	147.0	3.7 (2.9)	9.0 (5.8)
	Generalised tonic-clonic seizure	28	10.3 (7.1, 14.9)	10.2 (7.1, 14.8)	231.7	3.3 (2.8)	8.7 (6.4)
	Sudden death	21	10.8 (7.1, 16.6)	10.8 (7.1, 16.6)	186.8	3.4 (2.8)	8.7 (6.1)
	Thinking abnormal	20	10.9 (7.0, 16.9)	10.9 (7.0, 16.9)	179.8	3.4 (2.8)	8.7 (6.1)
>64	Emotional disorder	11	15.3 (8.5, 27.4)	15.3 (8.5, 27.3)	150.4	3.9 (3.1)	9.5 (6.0)
	Anger	17	12.6 (7.9, 20.3)	12.6 (7.8, 20.1)	183.5	3.6 (3.0)	9.4 (6.4)
	Status epilepticus	10	15.7 (8.5, 29.0)	15.7 (8.5, 28.9)	141.9	3.9 (3.1)	9.3 (5.7)
	Suicidal ideation	21	10.8 (7.0, 16.5)	10.7 (7.0, 16.4)	187.0	3.4 (2.8)	8.7 (6.2)
	Apathy	13	12.1 (7.1, 20.7)	12.0 (7.0, 20.6)	134.7	3.6 (2.8)	8.6 (5.6)

In patients under 18 years of age, significant safety signals were detected for tachycardia (EBGM = 8.3, EBGM05 = 5.3) and somnolence (EBGM = 7.9, EBGM05 = 5.5). Both events demonstrated high ROR and PRR values with narrow confidence intervals, suggesting robust associations in this age group.

Among patients aged 18–44 years, a strong cluster of psychiatric and metabolic adverse events was observed, including feeling guilty, drug withdrawal syndrome, restless legs syndrome, depersonalization/derealisation disorder, hypoglycemia, psychiatric symptoms, decreased interest, galactorrhoea, apathy, and gestational diabetes. The most prominent signals in this group were feeling guilty (EBGM = 9.7, EBGM05 = 6.2) and drug withdrawal syndrome (EBGM = 9.5, EBGM05 = 8.1), with high χ^2^ values (>800), indicating strong drug-event associations.

For the 45–64-year age group, notable events included shock hemorrhagic, bruxism, dependence, colitis microscopic, mydriasis, generalized tonic-clonic seizure, sudden death, and thinking abnormal. The highest signal intensity was found for shock hemorrhagic (EBGM = 9.9, EBGM05 = 6.3), followed closely by bruxism (EBGM = 9.4, EBGM05 = 6.0), both showing high ROR (15) and IC values (>3.8), denoting strong statistical significance.

In patients over 64 years of age, prominent PTs included emotional disorder, anger, status epilepticus, suicidal ideation, and apathy. The strongest associations were identified for emotional disorder (EBGM = 9.5, EBGM05 = 6.0), anger (EBGM = 9.4, EBGM05 = 6.4), and status epilepticus (EBGM = 9.3, EBGM05 = 5.7). All of these events presented elevated ROR and PRR (>12) and IC values greater than 3.5, reflecting consistent signal patterns in elderly patients.

### 3.6 Demographic characteristics of death and non-death cases

Table 6 presents the demographic characteristics of venlafaxine-related adverse event reports, comparing cases resulting in death with those that did not. A total of 2,660 death cases and 44,552 non-death cases were identified. Among death cases, female patients accounted for 1,569 (3.32%), representing a larger proportion compared to males (1,076, 2.28%), with the difference being statistically significant (P < 0.001).

**TABLE 6:** Characteristics between death outcomes and non-death outcomes.

Characteristic		Death cases, n (%)	Non-death cases, n (%)	P-value
Gender, n (%)	F	1,569 (3.32)	31,696 (67.14)	​
Gender, n (%)	M	1,076 (2.28)	12,425 (26.32)	<0.001
Gender, n (%)	N	15 (0.03)	336 (0.71)	​
Gender, n (%)	U	0 (0.00)	95 (0.20)	​
Age group, n (%)	<18	25 (0.05)	682 (1.30)	​
Age group, n (%)	18–44	1,121 (2.13)	13,226 (25.12)	2.806
Age group, n (%)	45–64	885 (1.68)	11,877 (22.56)	​
Age group, n (%)	>64	414 (0.79)	6,128 (11.64)	​
Age group, n (%)	Unknown	530 (1.01)	17,762 (33.74)	​

With respect to age distribution, death cases were predominantly reported in the 18–44 years (1,121 cases, 2.13%) and 45–64 years (885 cases, 1.68%) age groups. The >64 years group accounted for 414 cases (0.79%), while patients under 18 years represented only 25 cases (0.05%). A significant difference in age group distribution between death and non-death cases was observed (P = 0.0028). Overall, these results indicate that fatalities associated with venlafaxine were more frequently observed among adults aged 18–64 years, with a higher proportion occurring in female patients.

### 3.7 Functional enrichment analyses of venlafaxine-associated genes

To contextualize the detected safety signals biologically and pharmacologically, functional enrichment analyses were conducted using a curated set of venlafaxine-associated genes derived from established pharmacological and safety literature (SLC6A4, SLC6A2, HTR2A, HTR2C, MAOA, MAOB, TPH2, BDNF, FKBP5, CYP2D6, CYP2C19, and ABCB1). These genes encompassed primary therapeutic targets, downstream components of serotonergic signaling, ion channel–related safety mechanisms, and key enzymes involved in drug metabolism, thereby capturing both pharmacodynamic and pharmacokinetic determinants of venlafaxine response.

**FIGURE 4:**
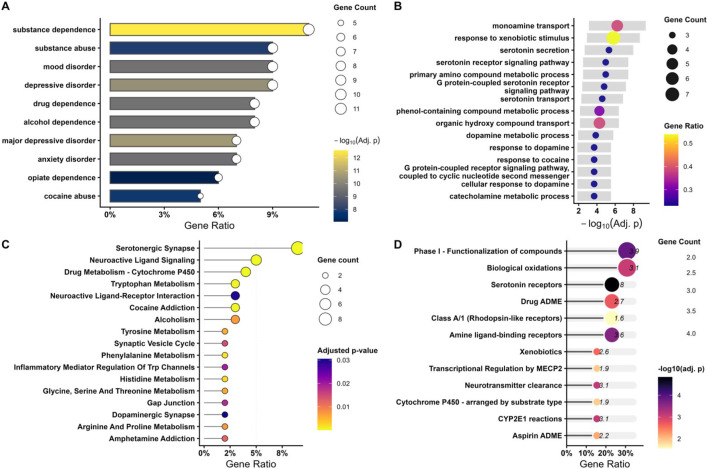
Functional analysis of venlafaxine targeted genes. Functional enrichment analyses of venlafaxine-associated genes. **(A)** Disease ontology enrichment analysis. **(B)** Gene ontology biological process analysis. **(C)** KEGG pathway analysis. **(D)** Reactome pathway analysis.

Disease Ontology enrichment demonstrated significant associations with mood disorder, depressive disorder, and anxiety disorders, providing clinical relevance to the strongest venlafaxine-related safety signals (Figure 4A). These disease-level annotations align with venlafaxine’s approved indications as a serotonin–norepinephrine reuptake inhibitor used in major depressive and anxiety disorders, supporting the interpretability of the FAERS-derived risk profile. Gene Ontology biological process analysis showed significant enrichment for monoamine transport, serotonin secretion, serotonin receptor signaling pathway, and dopamine response. These enriched processes are consistent with venlafaxine’s primary actions on monoaminergic neurotransmission and support the biological plausibility of neuropsychiatric adverse events identified in the FAERS analysis (Figure 4B).

KEGG pathway analysis indicated significant enrichment in serotonergic synapse signaling, neuroactive ligand–receptor interaction, synaptic vesicle cycle, and dopaminergic synapse pathways (Figure 4C). These findings suggest that venlafaxine-associated targets are functionally integrated within central nervous system neurotransmission and autonomic regulatory circuits, providing a mechanistic framework for both therapeutic effects and off-target safety signals. Reactome pathway analysis further revealed enrichment of neuronal system signaling pathways, serotonin receptor signaling, and cytochrome P450–mediated drug metabolism, highlighting convergent central pharmacodynamic effects and hepatic pharmacokinetic processes relevant to interindividual variability in adverse event susceptibility (Figure 4D). Collectively, these functional annotations support that the highest-intensity venlafaxine-associated adverse events, including suicide attempts and agitated depression, are underpinned by coherent pharmacological and biological mechanisms rather than isolated statistical artifacts.

## 4 Discussion

In this large-scale FAERS analysis of 47,325 venlafaxine-related adverse event reports from 2004 to 2025, several well-established safety concerns were confirmed. The strong signals for suicide attempts and completed suicide align with the FDA boxed warning on increased suicidal ideation in children, adolescents, and young adults (Coupland et al., 2015; Rynn et al., 2017; Kamp et al., 2024). While short-term RCTs have shown uncertain but potentially elevated risks of suicidality, our real-world data provide complementary evidence over a broader population and longer timeframe. Commonly documented adverse effects, including nausea, dizziness, somnolence, dry mouth, insomnia, and sexual dysfunction, were consistent with RCT and post-marketing literature (Parinda Parikh et al., 2025). Similarly, venlafaxine’s labeled risks for hypertension, serotonin syndrome, and withdrawal syndrome were supported, with signal detection emphasizing the need for monitoring, particularly in adults with comorbidities (Kıvrak et al., 2014; Calvi et al., 2021). These findings underscore the importance of confirming known safety issues in a large, heterogeneous population beyond the controlled trial environment.

Beyond known risks, our study revealed several less-characterized safety signals. “Drug ineffective” was the most frequently reported PT, emphasizing the clinical issue of treatment non-response, particularly in second-line or augmentation therapy. Although ‘drug ineffective’ was the most frequently reported PT, this term represents therapeutic non-response and does not constitute a biological adverse event. Its prominence underscores real-world treatment challenges but should not be interpreted alongside mechanistic safety signals. While RCTs assess efficacy, spontaneous reports rarely focus on non-response, suggesting that real-world pharmacovigilance can capture treatment gaps and guide early dose adjustments or switching strategies (Moncrieff et al., 2025). Additionally, rare but high-intensity signals such as renal dysplasia, agitated depression, and parotid gland enlargement may represent idiosyncratic reactions or off-target toxicities. While causality cannot be inferred, these findings suggest avenues for case-series reviews and mechanistic studies. Renal dysplasia is particularly notable given venlafaxine’s reduced clearance in renal impairment (Troy et al., 1994), warranting additional monitoring in this subgroup.

Age-stratified analyses revealed distinct safety patterns. In the <18-year cohort, tachycardia and somnolence were prominent, reflecting cardiovascular and central nervous system sensitivity in younger patients. Previous pediatric studies (45 patients) documented new agitation and suicidal ideation in approximately 6%–7% of cases, supporting our findings (Rynn et al., 2007; Simas et al., 2024). Among older adults (>64 years), emotional disorder, anger, and status epilepticus were notable, highlighting neurologic and psychiatric vulnerability in this population, consistent with prior reports of reduced tolerability versus other antidepressants in frail elderly patients (Oslin et al., 2003). These results emphasize the need for age-tailored monitoring strategies, particularly for neuropsychiatric and adverse cardiovascular events.

The bimodal time-to-onset distribution, with 39% of events occurring within 30 days and 21% beyond 360 days, indicates both early and late risk windows. Early events align with known tolerability issues such as nausea, somnolence, and anxiety. In contrast, late-onset events underscore the limitations of short-term RCTs (≤12 weeks) in capturing delayed adverse effects (Kamp et al., 2024). Hospitalization occurred in 52.7% of reports and deaths in 16.7%, highlighting the clinical significance of post-marketing events. Deaths were most frequent among adults aged 18–64 and slightly more common in females, emphasizing the need for careful risk–benefit assessment across demographic subgroups.

Female predominance in reports (63.8%) likely reflects both prescribing patterns and sex-specific susceptibility or reporting behavior. Strong signals for feelings of worthlessness and guilt in females suggest heightened vulnerability to mood-domain adverse events. Among males, high EBGM values for maternal and fetal exposure may reflect reporting nuances or misclassification but warrant further investigation. These sex-based differences highlight the need for individualized patient counseling and monitoring.

These enrichment results support a mechanistically grounded interpretation of the venlafaxine safety signals observed in FAERS, indicating that the most prominent adverse events arise within the same neurobiological and pharmacokinetic framework as the drug’s therapeutic actions. The convergence of Disease Ontology, Gene Ontology, KEGG, and Reactome findings highlighting mood and anxiety disorders, monoaminergic transport and signaling, serotonergic and dopaminergic synaptic pathways, autonomic regulation, and cytochrome P450–mediated metabolism suggests that signals such as suicide attempt and agitated depression are embedded in coherent biological networks rather than representing isolated statistical artifacts.

Taken together, this study extends current knowledge by confirming the consistency of known label warnings while identifying new or rarely described risks from two decades of real-world evidence. By integrating multiple disproportionality models across demographic subgroups, it contributes to a detailed understanding of venlafaxine’s safety landscape. Future research should prioritize mechanistic exploration of high-signal, low-frequency events and incorporate pharmacoepidemiological validation using linked electronic health record systems to overcome the limitations of spontaneous reporting data, such as underreporting and incomplete exposure detail.

Future validation studies could leverage large EHR platforms such as the FDA Sentinel Initiative, OMOP/OHDSI databases, MarketScan, Optum, or CPRD. These resources allow for verification of key safety signals such as suicidality, hypertensive crises, neonatal complications, and withdrawal phenomena using robust confounder adjustment methods, including active-comparator designs, propensity score weighting, and high-dimensional propensity scores.

Although congenital and neonatal signals (renal dysplasia, neonatal cerebral hemorrhage) were based on few reports, they represent clinically important findings that require follow-up in maternal–infant linked EHR datasets to differentiate signal from artifact.

In conclusion, these findings highlight the breadth of venlafaxine-associated safety signals in clinical practice, emphasizing both expected adverse effects and emerging risks across different age and sex groups. Long-term pharmacovigilance, coupled with personalized treatment strategies and proactive monitoring, remains essential to optimize the risk–benefit balance of venlafaxine therapy in major depressive disorder.

## 5 Limitations

The imitations include the underreporting of adverse events, inconsistencies in report accuracy, missing or incomplete information, various reporting biases, confounding by indication, and the inability to determine causality between drug exposure and the reported outcomes. Nevertheless, spontaneous reporting systems still offer meaningful real-world safety evidence, supplementing clinical trial data that are often constrained by small sample sizes, brief study durations, and limited representation of high-risk or diverse patient groups, including older adults, children, and individuals with multiple comorbid conditions. Because FAERS lacks consistent information on concomitant therapy, dosage, and time on treatment, the observed AE patterns cannot be interpreted in a dose-dependent or indication-specific manner. These missing exposure characteristics are inherent limitations of spontaneous reporting systems and restrict causal inference. Some implausible PTs, such as maternal or foetal exposure reported in males, likely arise from data-entry or linkage errors. These artifacts highlight the importance of cautious interpretation of subgroup signals.

## Data Availability

The original contributions presented in the study are included in the article/Supplementary Material, further inquiries can be directed to the corresponding authors.

## References

[B1] American Psychiatric Association (2022). Diagnostic and statistical manual of mental disorders, text revision. 5th ed. American Psychiatric Association.

[B2] Baldwin D. S. Montgomery S. A. Nil R. Lader M. (2007). Discontinuation symptoms in depression and anxiety disorders. Int. J. Neuropsychopharmacol. 10 (1), 73–84. 10.1017/S1461145705006358 16359583

[B3] Bekka E. Eyer F. (2022). Dose-related hypoglycemia in venlafaxine poisoning: a retrospective cohort study. Clin. Toxicol. 60 (12), 1336–1344. 10.1080/15563650.2022.2138762 36332110

[B4] Calvi A. Fischetti I. Verzicco I. Belvederi Murri M. Zanetidou S. Volpi R. (2021). Antidepressant drugs effects on blood pressure. Front. Cardiovasc. Med. 8, 704281. 10.3389/fcvm.2021.704281 34414219 PMC8370473

[B5] Cao B. Park C. Subramaniapillai M. Lee Y. Iacobucci M. Mansur R. B. (2019). The efficacy of vortioxetine on anhedonia in patients with major depressive disorder. Front. Psychiatry 10, 17. 10.3389/fpsyt.2019.00017 30766492 PMC6365446

[B6] Coupland C. Hill T. Morriss R. Arthur A. Moore M. Hippisley-Cox J. (2015). Antidepressant use and risk of suicide and attempted suicide or self-harm in people aged 20 to 64: cohort study using a primary care database. Bmj, 350. 10.1136/bmj.h517 25693810 PMC4353276

[B7] European Medicines Agency (2008). Venlafaxine. Available online at: https://www.ema.europa.eu/en/medicines/human/referrals/efexor.

[B8] Evans-Lacko S. A. Aguilar-Gaxiola S. Al-Hamzawi A. Alonso J. Benjet C. Bruffaerts R. (2018). Socio-economic variations in the mental health treatment gap for people with anxiety, mood, and substance use disorders: results from the WHO World Mental Health (WMH) surveys. Psychol. Med. 48 (9), 1560–1571. 10.1017/S0033291717003336 29173244 PMC6878971

[B9] Fervaha G. Foussias G. Takeuchi H. Agid O. Remington G. (2016). Motivational deficits in major depressive disorder: cross-sectional and longitudinal relationships with functional impairment and subjective well-being. ComprPsychiatry 66, 31–38. 10.1016/j.comppsych.2015.12.004 26995233

[B10] Global Burden of Disease (GBD) 2021 (2024). Global burden of disease. Seattle: Institute for Health Metrics and Evaluation. Available online at: https://vizhub.healthdata.org/gbd-results/. 10.1093/ije/dyae122 PMC11427672 39327917

[B11] Haddad P. M. Talbot P. S. Anderson I. M. McAllister-Williams R. H. (2015). Managing inadequate antidepressant responses in depressive illness. Br. Med. Bull. 115 (1), 183–201. 10.1093/bmb/ldv034 26311502

[B12] Hammad T. A. Laughren T. Racoosin J. (2006). Suicidality in pediatric patients treated with antidepressant drugs. Arch. General Psychiatry 63 (3), 332–339. 10.1001/archpsyc.63.3.332 16520440

[B13] Hieronymus F. (2019). Which antidepressant doses are optimal? Lancet Psychiatry 6 (7), 552–554. 10.1016/S2215-0366(19)30221-4 31178368

[B14] Holliday S. M. Benfield P. (1995). Venlafaxine: a review of its pharmacology and therapeutic potential in depression. Drugs 49 (2), 280–294. 10.2165/00003495-199549020-00010 7729333

[B15] Howell C. Wilson A. D. Waring W. S. (2007). Cardiovascular toxicity due to venlafaxine poisoning in adults: a review of 235 consecutive cases. Br. J. Clin. Pharmacol. 64 (2), 192–197. 10.1111/j.1365-2125.2007.02849.x 17298480 PMC2000637

[B16] Kaiser R. H. Andrews-Hanna J. R. Wager T. D. Pizzagalli D. A. (2015). Large-scale network dysfunction in major depressive disorder: a meta-analysis of resting-state functional connectivity. JAMA Psychiatry 72 (6), 603–611. 10.1001/jamapsychiatry.2015.0071 25785575 PMC4456260

[B17] Kamp C. B. Petersen J. J. Faltermeier P. Juul S. Siddiqui F. Moncrieff J. (2024). The risks of adverse events with venlafaxine for adults with major depressive disorder: a systematic review of randomized clinical trials with meta-analysis and trial sequential analysis. Epidemiol. Psychiatric Sci. 33, e51. 10.1017/S2045796024000520 39440379 PMC11561525

[B18] Kaplan E. M. (2002). Efficacy of venlafaxine in patients with major depressive disorder who have unsustained or no response to selective serotonin reuptake inhibitors: an open-label, uncontrolled study. Clin. Therapeutics 24 (7), 1194–1200. 10.1016/s0149-2918(02)80029-7 12182262

[B19] Kent J. M. (2000). SNaRIs, NaSSAs, and NaRIs: new agents for the treatment of depression. Lancet 355 (9207), 911–918. 10.1016/S0140-6736(99)11381-3 10752718

[B20] Khalifa M. Daleau P. Turgeon J. (1999). Mechanism of sodium channel block by venlafaxine in Guinea pig ventricular myocytes. J. Pharmacol. Exp. Ther. 291 (1), 280–284. 10.1016/s0022-3565(24)35098-0 10490914

[B21] Kıvrak Y. Güvenç T. S. Akbulut N. Yağcı İ G. Gündüz S. Balcı B. (2014). Accelerated hypertension after venlafaxine usage. Case Rep. Psychiatry 2014 (1), 659715. 10.1155/2014/659715 25328745 PMC4190979

[B22] Kobylianskii J. Wu P. E. (2021). Venlafaxine-induced hypoglycemia. Can. Med. Assoc. J. 193 (16), E568. 10.1503/cmaj.78409 33875464 PMC8084559

[B23] McGrath J. J. Lim C. C. Plana-Ripoll O. Holtz Y. Agerbo E. Momen N. C. (2020). Comorbidity within mental disorders: a comprehensive analysis based on 145 990 survey respondents from 27 countries. Epidemiol. Psychiatric Sci. 29, e153. 10.1017/S2045796020000633 32782057 PMC7443806

[B24] Moncrieff J. Hobday H. Sørensen A. Read J. Plöderl M. Hengartner M. (2025). Evidence on antidepressant withdrawal: an appraisal and reanalysis of a recent systematic review. Psychol. Med. 55, e191. 10.1017/S0033291725100652 40692314 PMC12315658

[B25] National Health Service (2018). Venlafaxine. Available online at: https://www.nhs.uk/medicines/venlafaxine/.

[B26] Oslin D. W. Have T. R. Streim J. E. Datto C. J. Weintraub D. DiFilippo S. (2003). Probing the safety of medications in the frail elderly: evidence from a randomized clinical trial of sertraline and venlafaxine in depressed nursing home residents. J. Clin. Psychiatry 64 (8), 875–882. 10.4088/jcp.v64n0804 12927001

[B27] Özdemir S. (2021). A case report of rare adverse events associated with venlafaxine administration: hypoglycemia and lactic acidosis. Korean J. Fam. Med. 43 (1), 83–85. 10.4082/kjfm.20.0100 34802219 PMC8820965

[B28] Parinda Parikh M. D. Mathew N. M. Narravula R. Eunsaem Lee M. D. Dilinuer Wubuli M. D. Isa Emre Gültekin M. D. (2025). Venlafaxine induced priapism in an adult male: a rare adverse effect. IJPS 7 (1), 09–11. 10.33545/26649241.2025.v7.i1a.20

[B29] Rynn M. A. Riddle M. A. Yeung P. P. Kunz N. R. (2007). Efficacy and safety of extended-release venlafaxine in the treatment of generalized anxiety disorder in children and adolescents: two placebo-controlled trials. Am. J. Psychiatry 164 (2), 290–300. 10.1176/ajp.2007.164.2.290 17267793

[B30] Rynn M. A. Yanes-Lukin P. K. Braun D. A. Goldberg P. H. (2017). Anxiety disorders begin in childhood and often evolve into adulthood, re. Clin. Man. Child Adolesc. Psychopharmacol.

[B31] Simas K. R. Reynolds L. Alnajjar A. Taweel Z. Almajarresh E. (2024). The tolerability and side effect profile of venlafaxine in a clinical outpatient pediatric population. J. Psychiatry Cogn. Behav. 7, 189. 10.29011/2574-7762.000089

[B32] Singh D. Saadabadi A. (2024). Venlafaxine. InStatPearls. Treasure Island, FL: StatPearls Publishing. 30570984

[B33] Sternbach H. (1991). The serotonin syndrome. Am. J. Psychiatry 148 (6), 705–713. 10.1176/ajp.148.6.705 2035713

[B34] Stewart W. F. Ricci J. A. Chee E. Hahn S. R. Morganstein D. (2003). Cost of lost productive work time among US workers with depression. JAMA 289 (23), 3135–3144. 10.1001/jama.289.23.3135 12813119

[B35] Stone M. Laughren T. Jones M. L. Levenson M. Holland P. C. Hughes A. (2009). Risk of suicidality in clinical trials of antidepressants in adults: analysis of proprietary data submitted to US Food and Drug Administration. Bmj, 339. 10.1136/bmj.b2880 19671933 PMC2725270

[B36] Troy S. M. Schultz R. W. Parker V. D. Chiang S. T. Blum R. A. (1994). The effect of renal disease on the disposition of venlafaxine. Clin. Pharmacol. and Ther. 56 (1), 14–21. 10.1038/clpt.1994.95 8033490

[B37] U.S. Food and Drug Administration (2017). Effexor XR (venlafaxine extended release) prescribing information. Silver Spring, MD: FDA.

[B38] USFDA (2009). Venlafaxine. Available online at: https://www.accessdata.fda.gov/drugsatfda_docs/nda/2008/022104_venlafaxine_hydrochloride_toc.cfm.

[B39] Vinetti M. Haufroid V. Capron A. Classen J. F. Marchandise S. Hantson P. (2011). Severe acute cardiomyopathy associated with venlafaxine overdose and possible role of CYP2D6 and CYP2C19 polymorphisms. Clin. Toxicol. 49 (9), 865–869. 10.3109/15563650.2011.626421 22077251

[B40] Woody C. A. Ferrari A. J. Siskind D. J. Whiteford H. A. Harris M. G. (2017). A systematic review and meta-regression of the prevalence and incidence of perinatal depression. J. Affect. Disord. 219, 86–92. 10.1016/j.jad.2017.05.003 28531848

