# Supplementary material for: The post-marketing safety of venlafaxine: a real-world two-decade pharmacovigilance study using the FAERS database
Source: Front Pharmacol. 2026 Jan 21;17:1737113. doi: 10.3389/fphar.2026.1737113 (PMC12868114; doi:10.3389/fphar.2026.1737113)
Supplement: Supplementary file 1 [file DataSheet1.pdf]

Sup. Table 1. The 2x2 contingency table for disproportionality analysis.

	**Target drug**	**Other drugs**	**Total**
Adverse events of interest	a	b	a+b
Other adverse events	C	D	C+d
Total	a+c	b+d	a+b+c+d

Sup Table 2. Signal detection algorithms, formulas, and established thresholds used in the analysis.

Algorithms	Equation	Threshold
ROR	ROR = $\frac{ad}{bc}$ $SE(lnROR) = \sqrt{\left(\frac{1}{a}+\frac{1}{b}+\frac{1}{c}+\frac{1}{d}\right)}$ $95\%\ CI = e^{lnROR \pm 1.96\sqrt{\frac{1}{a}+\frac{1}{b}+\frac{1}{c}+\frac{1}{d}}}$	N ≥ 3, lower limit of 95% CI > 1
PRR	$PRR = \frac{\frac{a}{a+b}}{\frac{c}{c+d}}$ $SE(lnPRR) = \sqrt{\frac{1}{a}-\frac{1}{a+b}+\frac{1}{c}-\frac{1}{c+d}}$ $95\%\ CI = e^{ln(PRR) \pm 1.96\sqrt{\frac{1}{a}-\frac{1}{a+b}+\frac{1}{c}-\frac{1}{c+d}}}$ $\chi^2 = \frac{[(ad - bc)^2(a + b + c + d)]}{[(a + b)(c + d)(a + c)(b + d)]}$	N ≥ 3, lower limit of 95% CI > 1, $\chi^2 \geq 4$
BCPNN	$IC = log_2\frac{a(a+b+c+d)}{(a+b)(a+c)}$ $\gamma = \frac{\gamma 11(a+b+c+d+\alpha)(a+b+c+d+\beta)}{(a+b+\alpha 1)(a+c+\beta 1)}$ $E(IC) = log_2\frac{(a+\gamma 11)(a+b+c+d+\alpha)(a+b+c+d+\beta)}{(a+b+c+d+\gamma)(a+b+\alpha 1)(a+c+\beta 1)}$ $V(IC) = \frac{1}{(ln2)^2}\left\{\frac{[(a+b+c+d)-a+\gamma-\gamma 11]}{[(a+\gamma 11)(1+a+b+c+d+\gamma)]}+\frac{[(a+b+c+d)-(a+b)+a-\alpha 1]}{[(a+b+\alpha 1)(1+a+b+c+d+\alpha)]}+\frac{[(a+b+c+d)-(a+c)+\beta-\beta 1]}{[(a+c+\beta 1)(1+a+b+c+d+\beta)]}\right\}$ $95\%\ CI = E(IC) \pm 2\sqrt{V(IC)}$	IC025 > 0
EBGM	$EBGM = \frac{a+b+c+d}{(a+c)(a+b)}$ $95\%\ CI = e^{ln(EBGM) \pm 1.96\sqrt{\frac{1}{a}+\frac{1}{b}+\frac{1}{c}+\frac{1}{d}}}$	EBGM05 > 2
